# Update in the management of chronic lymphocytic leukemia

**DOI:** 10.1186/1756-8722-2-29

**Published:** 2009-07-20

**Authors:** Kami J Maddocks, Thomas S Lin

**Affiliations:** 1 The Ohio State University, Division of Hematology-Oncology, 320 West 10^th ^Avenue, Columbus, Ohio 43210, USA

## Abstract

Advances in the treatment of chronic lymphocytic leukemia (CLL) have improved initial overall response (OR) rates, complete response (CR) rates and progression free survival (PFS). Despite these advances, CLL remains incurable with standard therapies. Thus, there remains a need for more effective therapies in both the upfront and relapsed setting, particularly for patients with high-risk cytogenetic abnormalities such as del(11q22) and del(17p13). The 2008 American Society of Hematology (ASH) Annual Meeting featured several presentations which highlighted the ongoing clinical advances in CLL. The benefit of adding rituximab to purine analog therapy in the upfront setting was demonstrated by a large randomized study which showed that the addition of rituximab to fludarabine and cyclophosphamide (FCR) significantly improved OR, CR and PFS. The improvement in PFS directly resulted from an improved ability to eliminate minimal residual disease (MRD) in the peripheral blood, highlighting the importance of MRD eradication. However, a multi-center study suggested that the high CR rates to chemoimmunotherapy regimens such as FCR obtained in academic centers may not be reproducible when the same regimens are given in the community setting. The immunomodulatory drug lenalidomide is active in relapsed high-risk CLL, but two studies of lenalidomide in previously untreated CLL patients failed to achieve a CR and were associated with significant tumor lysis, tumor flare and hematologic toxicity. In the relapsed setting, a combination study of the bifunctional alkylator bendamustine and rituximab (BR) demonstrated a high OR rate in patients with del(11q22) and del(17p13), indicating that further studies to define's bendamustine activity are warranted in high-risk CLL. Similarly, the CDK inhibitor flavopiridol demonstrated significant clinical activity and durable remissions in heavily treated, refractory CLL patients with high-risk cytogenetic features and bulky lymphadenopathy. The monoclonal anti-CD20 antibody ofatumumab appeared to be superior to rituximab in relapsed CLL patients with bulky nodal disease or high-risk cytogenetic features. Ongoing studies of these agents and other novel therapeutic agents in clinical development hold forth the promise that treatment options for CLL patients will continue to expand and improve.

## Introduction

Chronic lymphocytic leukemia (CLL) is the most common adult leukemia in the Western hemisphere. Although patients with early stage disease have a greater than 10 year life expectancy, patients with more advanced disease have a median survival of 18 months to 3 years and those who have fludarabine refractory disease have a median survival of less than one year. Advances in the therapy for CLL, particularly "chemoimmunotherapy" regimens combining cytotoxic agents such as alkylating agents and purine nucleoside analogs with monoclonal antibodies such as rituximab, have improved initial overall response (OR) rates, complete response (CR) rates and progression free survival (PFS). Despite these advances, CLL remains incurable with standard therapies; patients inevitably relapse, become increasingly refractory to treatment, and often acquire high-risk chromosomal abnormalities such as del(11q22) and del(17p13), which correspond to loss of the *ataxia telangiectasia mutated *(ATM) and p53 tumor suppressor genes, respectively. Thus, there remains a need for more effective therapies in both the upfront and relapsed setting. This review highlights advances in the treatment of CLL in both previously untreated and relapsed disease and focuses on new data presented at the 2008 American Society of Hematology (ASH) Annual Meeting.

## Background studies

### Indications for therapy

The 1996 NCI Working Group established guidelines for initiating treatment for CLL, which were affirmed in 2008 by the International Workshop on CLL[1,2]. Indications include autoimmue and non-autoimmune cytopenias, bulky or symptomatic lymphadenopathy or organomegaly, disease-related B-symptoms or fatigue, and rapid lymphocyte doubling time. Since CLL is typically diagnosed by routine complete blood count examination, most patients are asymptomatic without cytopenias at diagnosis. Asymptomatic patients should be followed expectantly and should receive treatment only upon disease progression. While ongoing, prospective studies such as the Cancer and Leukemia Group B (CALGB) 10501 trial are studying whether early intervention affects long-term outcome of patients with high-risk features such as unmutated IgVH, there currently are no data supporting early therapy for high-risk CLL patients who do not meet criteria for therapy. Any early treatment intervention for asymptomatic high-risk patients should only be performed in the setting of a clinical study. Thus, it is important to determine that all patients required therapy by NCI and IWCLL criteria when evaluating results of upfront clinical studies in CLL. Of note, many studies in previously untreated CLL focus on the CR rate and PFS, whereas overall survival (OS) remains the most meaningful endpoint of any therapeutic cancer trial. Nonetheless, CR and PFS are important measures of a treatment's efficacy, as patients who achieve CR and enjoy prolonged PFS are likely to experience improved quality of life. Furthermore, identification of regimens which achieve high CR rates and durable PFS will provide insight for investigators to develop more effective future treatment strategies.

### Fludarabine combined with alkylator therapy

Three large, prospective, randomized, multi-center studies showed that combination therapy with fludarabine and cyclophosphamide (FC) is superior to fludarabine alone with respect to OR, CR, and PFS in previously untreated CLL patients (Table 1). However, there was no benefit in OS in any of these studies. The German CLL Study Group (GCLLSG) randomized 375 patients to fludarabine 25 mg/m^2 ^intravenously (IV) daily for 5 days versus fludarabine 30 mg/m^2 ^IV and cyclophosphamide 250 mg/m^2 ^IV daily for 3 days every 28 days for 6 cycles [3]. This study showed improved OR (94% vs. 83%), CR (24% vs. 7%), and PFS (48 vs. 20 months) in favor of FC. ECOG randomized 278 patients to fludarabine 25 mg/m^2 ^IV daily for 5 days versus fludarabine 20 mg/m^2 ^IV daily for 5 days and cyclophosphamide 600 mg/m^2 ^IV day 1 every 28 days for 6 cycles [4]. Results favored FC with respect to OR (74% vs. 59%), CR (23% vs. 5%), and PFS (32 vs. 19 months). Lastly, the United Kingdom CLL4 study randomized 777 patients to oral chlorambucil, fludarabine, or FC [5]. Again, patients receiving FC achieved superior OR (94% vs. 80%), CR (38% vs. 15%), and 5-year PFS (36% vs. 10%). Importantly, the study showed that patients with del(11q22) fared better with FC, but that patients with del(17p13) did poorly regardless of the treatment arm.

**Table 1:** Selected Trials in Previously Untreated CLL Patients

Reference	Regimen	Phase	No. Pts.	CR, %	ORR, %	Median PFS (months)
Eichhorst, 2006	Flu	III	164	7	83	20
	Flu + Cy		164	24	94	48
Flinn, 2007	Flu	III	137	5	59	19
	Flu + Cy		141	23	74	32
Catovsky, 2007	Chl	III	387	7	72	Not Rep
	Flu		194	15	80	Not Rep
	Flu + Cy		196	38	94	Not Rep
Byrd, 2003	FR (C)	Rand. II	51	47	90	NR
	FR (S)		53	28	77	NR
Keating, 2005	FCR	II	300	72	94	NR
Hallek, 2008	FC	III	409	23	85	32
	FCR		408	45	93	43
Kay, 2007	PCR	II	64	41	91	31
Kay, 2008	PR	II	33	30	79	12
Reynolds, 2008	PCR	III	92	7	45	Not Rep
	FCR		92	17	58	Not Rep

Key: No-Number; Flu-fludarabine; CLB-Chlorambucil; Cy-cyclophosphamide; Ritux-rituximab; FR (C) – fludarabine + rituximab, concurrent; FR (S) – fludarabine + rituximab, sequential; FCR – fludarabine, cyclophosphamide, rituximab; PCR – pentostatin, cyclophosphamide, rituximab; PR – pentostatin + rituximab; CR – complete response rate; ORR – overall response rate; PFS – progression free survival; NR – not reached; Not Rep – Not reported

### Chemoimmunotherapy

The addition of the monoclonal anti-CD20 antibody rituximab to purine analog based therapy also increased OR, CR and PFS without benefit in OS, as summarized in Table 1. The CALGB 9712 study randomized 104 patients to fludarabine with sequential or concurrent rituximab [6]. Patients received fludarabine 25 mg/m^2 ^days 1–5 every 28 days for 6 cycles with or without rituximab 375 mg/m^2 ^on day 1 of cycles 1–6 and day 4 of cycle 1. All patients received rituximab 375 mg/m^2 ^weekly for 4 doses beginning 2 months after completion of fludarabine. Patients in the concurrent FR arm had superior OR (90% vs. 77%) and CR (47% vs. 28%), but there was no difference in PFS between the two arms. The MD Anderson Cancer Center (MDACC) demonstrated superior results with the combination of fludarabine, cyclophosphamide and rituximab (FCR). The MDACC treated 300 patients with fludarabine 25 mg/m^2 ^and cyclophosphamide 250 mg/m^2 ^on days 2–4 of cycle 1 and days 1–3 of cycles 2–6, and rituximab 375 mg/m^2 ^on day 1 of cycle 1 and 500 mg/m^2 ^on day 1 of cycle 2–6, every 28 days for 6. OR was 94%, CR was 72%, 4-year relapse free survival (RFS) was 77%, and 4-year OS was 83% [7]. The impact of poor risk cytogenetic factors on response to FCR was not reported.

While FR and FCR achieved excellent results, the median age of patients in these studies was 63 and 58 years, respectively, in contrast to a median age of 72 at first therapy in the SEER database. A phase II study of PCR in 64 previously untreated CLL patients, including 18 ≥ age 70 years, indicated that older patients tolerate and respond to PCR [8,9]. Patients received pentostatin 2 mg/m^2 ^on day 1, cyclophosphamide 600 mg/m^2 ^on day 1, and rituximab 375 mg/m^2 ^on day 1 (100 mg/m2 on day 1 and 375 mg/m^2 ^on day 3 and 5 of cycle 1) every 21 days for up to 6 cycles. PCR was well tolerated. OR was 91%, CR was 41%, and median PFS was 33 months (Table 1). The ability to achieve CR was independent of del(11q22), but all 3 patients with del(17p13) failed to achieve a CR.

## New data presented at the 2008 ASH Annual Meeting

### Chemoimmunotherapy

Based on the above background studies, it is clear that adding cyclophosphamide to fludarabine in the upfront setting improves OR, CR and PFS in CLL. Kay and colleagues recently examined whether the addition of an alkylating agent to other purine nucleoside analogs similarly improves outcomes [10]. The Mayo Clinic and Ohio State retrospectively compared results of a phase II study of pentostatin and rituximab (PR) to previously published results using pentostatin, cyclophosphamide and rituximab (PCR) [8]. The pentostatin dose was increased to 4 mg/m^2 ^in the PR regimen, but demographics of patients in both studies were similar. OR and CR rates were similar for PR (79%, 30%) and PCR (91%, 41%), but median PFS was significantly shorter for PR (12 months) compared to PCR (31 months). These results supported previous findings, described above, that the addition of cyclophosphamide to fludarabine improves OR, CR and PFS (Table 1).

While excellent results were obtained with FR and FCR in phase II studies, the benefit of adding rituximab to a purine nucleoside analog has not been examined in a prospective randomized trial. Therefore, the GCLLSG CLL8 study randomized 817 previously untreated patients to fludarabine 25 mg/m^2 ^days 1–3 and cyclophosphamide 250 mg/m^2 ^days 1–3, with or without rituximab 500 mg/m^2 ^day 1 (375 mg/m^2 ^day 1 of cycle 1) [11]. OR, CR and median PFS favored FCR (93%, 45%, 43 months) over FC (85%, 23%, 32 months), although 2-year OS (91% vs. 88%) was similar (Table 1). The MDACC FCR trial previously showed that the ability to achieve ≤ 1% residual CLL by two-color flow cytometry significantly affected relapse free survival (RFS) and OS [7]. Five of 138 patients (4%) whose post-FCR bone marrow flow cytometry had ≤ 1% residual CLL relapsed, in contrast to 17 of 62 patients (27%) with > 1% residual CLL. The importance of eradicating minimal residual disease (MRD) was confirmed by the GCLLSG CLL8 trial, which demonstrated that median PFS depended upon the ability to eradicate MRD in the peripheral blood, with PFS increasing from 15 months (MRD ≥ 10^-2^) to 34 months (10^-4 ^≥ MRD > 10^-2^) to not reached (MRD < 10^-4^) with increasing eradication of MRD [12]. Furthermore, 67% of patients receiving FCR achieved MRD < 10^-4^, compared to only 34% of FC patients, thus accounting for the improved PFS with FCR. In addition to reaffirming the importance of eradicating MRD, the GCLLSG CLL8 study demonstrated that MRD can be determined from peripheral blood and that bone marrow biopsies are not necessary to gain MRD information.

While regimens such as FR, FCR and PCR have achieved excellent results in academic centers, there are limited data on whether such regimens are able to achieve similar results in the community setting. Therefore, US Oncology performed a multicenter, community-based trial that randomized 184 patients (80% previously untreated, 20% relapsed) to PCR or FCR, using the Memorial Sloan Kettering PCR regimen (pentostatin dose 4 mg/m^2^) given for 8 cycles and the Johns Hopkins FCR regimen (fludarabine 20 mg/m^2 ^days 1–5, cyclophosphamide 600 mg/m^2 ^day 1) [13]. The primary endpoint, incidence of grade 3–4 infections, was similar for PCR (34%) and FCR (31%). Only 50% of patients in both arms completed therapy, resulting in surprisingly low OR and CR rates for PCR (45%, 7%) and FCR (58%, 17%), as summarized in Table 1. While the study was not powered to show a statistically significant difference between FCR and PCR, these findings indicated that results from academic centers may not necessarily be reproducible in the community. Specifically, a reduced ability to complete planned treatment may reduce the clinical efficacy of these regimens outside academic centers.

Because of the ability of the anti-CD52 alemtuzumab (Campath-1H) to eradicate MRD and its activity in high-risk CLL patients, the MDACC added alemtuzumab to FCR, to determine if the efficacy of FCR could be improved [14]. The CFAR regimen consists of fludarabine 25 mg/m^2 ^on days 2–4, cyclophosphamide 250 mg/m^2 ^on days 2–4, rituximab 375 mg/m^2 ^(cycle 1) or 500 mg/m^2 ^(cycles 2–6) on day 2, and alemtuzumab 30 mg IV on days 1, 3 and 5 every 28 days for up to 6 cycles. Patients receive pegfilgrastrim, as well as prophylaxis for *pneumocystis carinii *pneumonia (PCP) and cytomegalovirus (CMV). A phase II study in 78 relapsed CLL patients had attained OR 65% (CR 24%); median PFS was 27 months for the 19 CR patients and 10 months for the 32 PR patients. Given these promising results, the MDACC is conducting a phase II study of CFAR in previously untreated patients [15]. Results in the first 48 patients with high-risk features revealed OR and CR of 94% and 69%, respectively, with OR 77% and CR 54% in 13 patients with del(17p13). Grade 3–4 neutropenia and thrombocytopenia were observed in 71% and 42% of patients, respectively, and 6% and 27% of patients developed major and minor infections, respectively. While the results do not appear to be superior to FCR at first glance, it must be noted that CFAR is being tested in higher risk patients and that PFS data are not yet available.

### Novel agents

Bendamustine hydrochloride is a novel alkylating agent which contains a benzimidazole ring and is only partially cross-resistant with other alkylating agents *in vitro*. The FDA approved bendamustine for the treatment of CLL in 2008 based upon a multi-center, European phase III study which randomized 319 previously untreated CLL patients to oral chlorambucil 0.8 mg/kg on day 1 and 15 every 28 days or bendamustine 100 mg/m^2 ^IV on day 1 and 2 every 28 days [16]. Treatment in both arms was given for up to 6 cycles or until disease progression. The results of this study were updated at the 2008 ASH meeting. OR, CR and median PFS favored bendamustine (67%, 32%, 21.5 months) over chlorambucil (30%, 2%, 8.3 months), although bendamustine caused greater hematologic toxicity (40% vs. 19%), especially grade 3–4 neutropenia (23% vs. 9%) [17]. The GLLSG is currently conducting a randomized trial in previously untreated patients to compare the combination of bendamustine and rituximab (BR) to the MDACC regimen of FCR, which has achieved the highest CR rate of any upfront CLL regimen to date.

Lenalidomide is an immunomodulatory drug that is a more potent analog of thalidomide. Lenalidomide has shown activity in relapsed CLL patients including activity in patients with high-risk cytogenetic features. The Roswell Park Cancer Insititute conducted a phase II study which administered lenalidomide 25 mg daily orally on days 1–21 every 28 days to 45 patients with relapsed CLL [18]. Results showed OR and CR rates of 47% and 9%, and responses were observed in fludarabine refractory patients and those with poor-risk 11q22 and 17p13 deletions. The MDACC chose a different dosing strategy and administered lenalidomide by continuous low dose at 10 mg orally daily, with a 5 mg dose escalation every 28 days up to a maximum dose of 25 mg daily, to 44 patients with relapsed CLL with 10 mg being the median delivered dose [19]. OR and CR rates of 32% and 7% were observed, and the OR rate was 31% in patients with high-risk cytogenetic abnormalities. However, tumor lysis and tumor flare reactions are potentially serious toxicities of lenalidomide, and the optimal dosing schedule with respect to safety and efficacy are undefined.

Two studies of lenalidomide in previously untreated patients were presented at the 2008 ASH meeting. Chen et al. summarized results of a phase I study in 25 Canadian patients [20]. Due to grade 5 sepsis and grade 3–4 tumor lysis, the dose was decreased from 25 mg to 2.5 mg and then escalated to 10 mg daily for 21 days every 28 days. Toxicity included fatigue (78%), tumor flare (78%), rash (48%) and grade 3–4 neutropenia (43%). OR and CR were 65% and 0%, respectively. The MDACC presented a study in 43 elderly patients age 65 years or older [19]. Lenalidomide was given continuously, and 5–10 mg daily was the median delivered dose. Grade 3–4 myelosuppression and tumor flare were observed in 26% and 44% of patients, respectively. OR and CR were 54% and 0%, respectively. While lenalidomide is clearly active in CLL, the absence of CR in previously untreated patients was disappointing.

Finally, James et al. presented a phase II study giving high dose methylprednisolone 1000 mg/m^2 ^day 1–3 every four weeks and weekly rituximab (total dose 4500–6750 mg/m^2^) to 28 previously untreated patients [21]. OR and CR were 96% and 32%, respectively. Patients with less prominent splenomegaly and lower beta-2-microglobulin levels were more likely to respond.

### Relapsed CLL

Despite advances in first line therapy for CLL, patients invariably relapse and often acquire high risk chromosomal abnormalities such as del(11q22) and del(17p13), which result in resistance to therapy [22,23]. Patients who have fludarabine refractory disease have a median survival of less than one year. Thus, new agents are needed for the treatment of relapsed CLL, particularly for those patients with high-risk cytogenetic features. It is important to emphasize that the same NCI and IWCLL criteria for initiating therapy in previously untreated patients also apply for patients with relapsed CLL [1,2]. Asymptomatic relapsed patients should be followed expectantly and should receive treatment only upon development of cytopenias or symptoms.

Stilgenbauer et al. presented final results of the GCLLSG CLL2H study which administered subcutaneous alemtuzumab to 103 relapsed patients, many of whom had high-risk features [24]. Infusion toxicity was minimal, but grade 3–4 anemia (56%), thrombocytopenia (57%), anemia (49%), cytomegalovirus reactivation (8%) and non-CMV infection (29%) were significant toxicities. Seventy-five patients died; 56% died of progressive CLL, and 31% died of infection. OR (34%), CR (4%) and median PFS (7.7 months) were similar to the results achieved by IV alemtuzumab in the pivotal CAM211 study [25]. While alemtuzumab was an effective therapy, the outcome for high-risk patients remained poor without the use of allogeneic stem cell transplantation.

The GCLLSG presented a phase II trial administering bendamustine 70 mg/m^2 ^on day 1–2 and rituximab 500 mg/m^2 ^on day 1 to 81 relapsed CLL patients [26]. OR and CR were 77% and 15%, respectively. Twelve of 13 patients (92%) with del(11q22), 4/9 patients (44%) with del(17p13), and 29/39 patients (74%) with unmutated IgVH responded, suggesting that bendamustine may be active in high-risk relapsed CLL. However, further studies are needed to determine whether bendamustine can be considered as a treatment for relapsed CLL patients with poor-risk features.

Flavopiridol is a synthetic flavone which broadly inhibits cyclin dependent kinases (CDK); down-regulates expression of anti-apoptotic proteins such as Mcl-1 and X-linked inactivator of apoptosis (XIAP); decreases phosphorylation and transcriptional activity of RNA polymerase II, resulting in decreased gene transcription; and induces apoptosis distally to p53 by activating caspase 3 in primary CLL cells. Lin et al. presented combined phase I/II results of flavopiridol (alvocidib) in 116 relapsed patients treated at Ohio State, 70% of whom were fludarabine-refractory [27]. OR in this high-risk population was 47%. Furthermore, 19/39 del(17p13) patients (49%), 28/47 del(11q22) patients (60%) and 22/52 complex karyotype patients (42%) responded, demonstrating the activity of flavopiridol in poor-risk groups with limited therapeutic options. Forty-one of 85 patients (48%) with bulky lymphadenopathy > 5 cm responded. Median PFS in responders was 10–12 months across all risk groups. Based upon these promising results, a registration study in 165 relapsed CLL patients is ongoing in the United States and Europe.

Several monoclonal anti-CD20 antibodies which have been engineered to improve antibody-dependent cytotoxicity or complement fixation are under clinical investigation. Of this new generation of anti-CD20 antibodies, ofatumumab has generated the most mature clinical data. Ofatumumab (HuMax-CD20) is a fully humanized, high-affinity monoclonal antibody whose epitope on the CD20 molecule of B cells is distinct from that of rituximab. Ofatumumab has higher affinity for CD20 than rituximab, activates complement-dependent cytotoxicity more effectively, and is superior to rituximab in killing B-cell lines with low CD20 expression. An initial phase I/II study in 33 patients with relapsed CLL giving weekly therapy for 4 week showed a 50% OR [28]. At the 2008 ASH meeting, Osterberg et al. presented a pivotal phase II study of ofatumumab in relapsed patients refractory to both fludarabine and alemtuzumab (DR, n = 59) or with bulky lymphadenopathy refractory to fludarabine (BFR, n = 79) [29]. OR, time to next therapy, and OS were similar for the DR (51%, 9.0 months, 13.7 months) and BFR groups (44%, 7.9 months, 15.4 months). Based on these results, ofatumumab has been submitted for FDA approval.

### Investigational Agents

Several exciting novel therapeutic agents are under study, although a complete description of these drugs is beyond the scope of this review. ABT-263, a small molecule inhibitor of Bcl-2, has shown clinical activity as a single agent in CLL, and studies of this drug in combination with other agents are ongoing. Based on the prior results with flavopiridol, several new CDK inhibitors are under clinical study. The MDACC has studied SNS-032, an inhibitor of CDK 2, 7 and 9. SCH 727965 is a CDK inhibitor which appears to have a better therapeutic window than flavopiridol, and this drug is under study in relapsed CLL as well as non-Hodgkin's lymphoma and multiple myeloma. The CD37 small molecule inhibitor (SMIP), Tru-016, is under investigation in relapsed CLL, and initial results of this study will be presented at the 2009 ASCO meeting. Similarly, preclinical results of an ongoing phase I study of CAL-101, an orally available inhibitor of the phosphatidylinositol-3-kinase (PI-3-K) delta isoform, will be presented at the ASCO meeting. Other novel agents are under preclinical and clinical study, but space precludes a discussion of these agents in their review.

## A Risk Stratified Approach

Fluorescence in situ hybridization (FISH) to determine cytogenetic abnormalities is now the standard of care in CLL; any practicing hematologist or oncologist has access to commercial laboratories which can perform adequate FISH testing. FISH should be performed before making treatment decisions in the upfront or relapsed setting, and it is important to remember that patients will acquire increasing chromosomal abnormalities in their CLL cells as their disease becomes more advanced over time. For previously untreated patients with del(11q22), FC or FCR should be considered in light of randomized studies showing marked improvement in CR and PFS with the addition of cyclophosphamide to fludarabine [3-5]. Unfortunately, there remains no standard of care for previously untreated patients with del(17p13), and these patients should be considered for non-myeloablative allogeneic stem cell transplantation after first treatment. Similarly, patients with del(17p13) are the largest therapeutic challenge in the relapsed setting, particularly given the increasing frequency of del(17p13) in relapsed patients. Alemtuzumab is active in these patients, although patients should be observed closely for hematologic and infectious toxicity [25]. Flavopiridol and lenalidomide are promising, but neither drug is approved for CLL [18,27,30]. Sadly, allogeneic stem cell transplantation remains the only therapy which offers prolonged PFS in these high-risk del(17p13) patients.

## Selecting a Treatment Regimen

Chemoimmunotherapy regimens such as FCR have achieved OR and CR rates in excess of 90% and 70%, respectively, and improved PFS in previously untreated CLL [7]. However, no OS advantage has been demonstrated for such regimens, due to similar improvements in treatment of relapsed CLL. Furthermore, patients in the community are generally older and in poorer medical condition than patients enrolled in upfront clinical studies at academic centers. Additionally, the goal of therapy may be different in older patients with multiple co-morbid medical conditions, in whom palliation of symptoms may be more important, than in younger patients in excellent health who hope to maximize their likelihood of long-term survival. Finally, risk stratification by cytogenetic features allows hematologists to identify patients, such as those with del(17p13), who require more aggressive treatment. Thus, there is no "best" initial therapy for CLL; treatment should be selected for an individual patient after considering the above points. Chlorambucil is still a reasonable option in elderly patients with multiple co-morbid illnesses who are poor candidates for fludarabine or in whom the primary goal is palliation. PCR is an alternative option for older patients in whom more aggressive therapy is desired [9]. Younger patients, particularly those who possess high-risk cytogenetic features, should be considered for a chemoimmunotherapy regimen such as FR or FCR [6,7]. Achievement of MRD negative marrow should be a therapeutic goal in younger, poor-risk patients, since eradication of MRD results in improved survival [12]. Unfortunately, patients with del(17p13) remain a therapeutic challenge, and non-myeloablative allogeneic stem cell transplantation should be considered for these high-risk patients.

## Conclusion

Several important results regarding therapy of CLL in both the upfront and relapsed settings were presented at the 2008 ASH Annual Meeting. The addition of cyclophosphamide or rituximab to purine analogs improves OR, CR and PFS, but no benefit in overall survival has been documented to date. Eliminating minimal residual disease (MRD) improves PFS, and MRD can be assessed from peripheral blood without the need for additional bone marrow biopsies. Despite the high CR rates reported with chemoimmunotherapy regimens in previously untreated CLL patients in academic centers, these results may not be reproducible in the community setting, due at least in part to decreased treatment delivery in the community. Bendamustine is active in both the upfront and relapsed setting, although hematologic toxicity requires a lower dose to be used in relapsed patients. Preliminary results of a phase II study suggest that the combination of bendamustine and rituximab (BR) may have activity in relapsed patients with high-risk cytogenetic features, but further studies are needed to determine whether bendamustine is an effective therapy for high-risk, relapsed CLL. Lenalidomide shows significant activity in high-risk relapsed CLL, but has limited ability to achieve a CR in the upfront setting and is associated with risks of tumor lysis, tumor flare and hematologic toxicity. The CDK inhibitor flavopiridol also demonstrates considerable activity against high-risk CLL, including bulky lymph node disease, but is associated with a risk of tumor lysis. The fully humanized anti-CD20 antibody ofatumumab appears to be superior to rituximab in relapsed CLL patients with bulky nodal disease or high-risk cytogenetic features. Ongoing studies of these agents and multiple other novel therapeutic agents in various stages of clinical development hold forth the promise that treatment options for CLL patients will continue to expand and improve with further clinical studies.

## Competing interests

The authors declare that they have no competing interests.

## Authors' contributions

KJM and TSL contributed equally to the research and writing of this manuscript. Both authors read and approved the final manuscript.
